# Formononetin Alleviates MNNG-Triggered Chronic Atrophic Gastritis: Its Potential Mechanisms

**DOI:** 10.2174/0113862073409559250618212035

**Published:** 2025-07-03

**Authors:** Yuling Wei, Wenhui Wu, Min Duan, Ting Li, Mei Liu, Jinyan Li

**Affiliations:** 1 Department of Pharmacy, Chongqing Hospital of Traditional Chinese Medicine, Chongqing 400000, China;; 2 Department of Gastroenterology, Chongqing Hospital of Traditional Chinese Medicine, Chongqing 400000, China;; 3 Department of Obstetrics and Gynecology, Chongqing Hospital of Traditional Chinese Medicine, Chongqing 400000, China

**Keywords:** Formononetin, chronic atrophic gastritis, inflammation, apoptosis, activator protein-1, nuclear receptor coactivator 1

## Abstract

**Introduction:**

Chronic atrophic gastritis (CAG) is the initial phase in the carcinogenesis of gastric cancer (GC). Therefore, effective treatment for CAG is important in reducing the risk of GC progression. As an isoflavone compound, formononetin (FMN) has been identified as a potential therapeutic agent for acute gastric ulcers and GC. However, no study has reported the protective effect of FMN against CAG and its underlying mechanism. This study aimed to explore the therapeutic effects of FMN on CAG and its underlying mechanisms *in vitro.*

**Methods:**

Network pharmacology was applied to predict the core targets of FMN therapy in CAG. The CAG cell model was developed using N-methyl-N’-nitro-N-nitrosoguanidine (MNNG)-triggered human gastric epithelial cells (GES-1). The CCK-8 assay was applied to estimate cellular viability. The expression of inflammatory cytokines in cell supernatant was detected by ELISA. The protein levels and localization of nuclear receptor coactivator 1 (NCOA1), c-Jun, and c-Fos were evaluated using western blotting and immunofluorescence staining. Cell apoptosis was measured using flow cytometry.

**Results:**

Network pharmacology analysis identified c-Jun as the core target of FMN in the treatment of CAG, with biological processes primarily involving the regulation of apoptosis and inflammation. *In vitro*, MNNG exposure reduced GES-1 cell viability as well as increased inflammation and cellular apoptosis, and these effects were reversed by FMN treatment. In detail, FMN decreased the protein levels of NCOA1, c-Jun, and c-Fos in MNNG-triggered GES-1 cells. The activator protein-1 (AP-1) inhibitor T-5224 enhanced the effects of FMN treatment on cell viability, inflammatory response, and apoptosis in MNNG-triggered GES-1 cells.

**Discussion:**

This study employed network pharmacology analysis to identify FMN's therapeutic targets for CAG and validated the underlying mechanisms *in vitro*. While these results are promising, *in vivo* validation is required to confirm the efficacy of FMN. A comparative pharmacological evaluation against existing therapeutic agents and bioactive compounds would further elucidate FMN's therapeutic potential for CAG treatment.

**Conclusion:**

FMN ameliorated the cell damage that MNNG triggered in GES-1 cells. The mechanism involved the anti-inflammatory and anti-apoptotic effects of FMN *via* modulation of the NCOA1/AP-1 signaling axis. The present preliminary study found FMN to exhibit a potential therapeutic effect against CAG.

## 1 INTRODUCTION

Globally, chronic atrophic gastritis (CAG) is a common, severe, and insidious inflammatory digestive disorder that arises from chronic gastritis. A systematic review revealed the prevalence of CAG in the study population to be approximately 25% [1]. CAG is distinguished by epithelial atrophy, a thin stomach mucosa, damage of gastric glands secreting mucus, and a thickened mucosal base, often accompanied by intestinal metaplasia and dysplasia, resulting from long-term disruption to the normal gastric cell structure due to inflammation [2]. Correa's pathway theory suggests that CAG is a crucial pathogenic phase in the transition from a normal condition to gastric cancer (GC) [3]. The yearly occurrence of GC in individuals with CAG is about 0.1%. After a follow-up of 1, 5, and 10 years after early diagnosis, GC is identified in 0.3%, 0.6%, and 0.8% of individuals, respectively [4]. A meta-analysis showed CAG patients to possess a 2–4-fold greater risk of developing GC, esophageal cancer, and esophagogastric junction malignancies [5]. Prompt intervention for CAG may avert the onset of GC and has considerable clinical importance. It is now believed that CAG has various causes, including bile reflux, *Helicobacter pylori*-induced infection, aging, and unhealthy diet [2]. Contemporary medicine predominantly employs non-specific therapies for CAG, encompassing the elimination of *Helicobacter pylori*, administration of antacids, and protection of mucosa [6]. However, CAG is prone to recurrence and is difficult to cure. Despite the increasing progress in pathological features of CAG and the discovery of curative targets, research on effective medications for CAG is still scarce [7]. Therefore, the exploration of novel medications for CAG is of significance [8].

Formononetin [FMN; 7-hydroxy-3-(4-methoxyphenyl) chromen-4-one], a member of the isoflavone family, is present in various species of the Fabaceae family, including red clover (*Trifolium pratense L.*) and *Astragalus membranaceus* (Fisch). Bunge [9]. With increasing research, it has been indicated that FMN has anti-apoptotic and anti-inflammatory properties [10]. In addition, increasing evidence has shown that a variety of bioactivities of FMN are associated with osteogenic, neuroprotective, antimicrobial, anticarcinogenic, and antihypertensive effects [11-15]. Previous studies have reported that FMN is efficient in preventing and treating tumors and gastrointestinal diseases, and it exhibits minimal or negligible toxicity in the process of treatment [16]. Mendonça *et al*. found that FMN mitigated gastric mucosal injury caused by ethanol and indomethacin [17]. Yi *et al*. demonstrated that FMN inhibited inflammation in rats with gastric ulcers by modulating NF-κB signaling [18]. However, the effects of FMN in CAG treatment are still unclear. Therefore, we hypothesized that FMN may ameliorate CAG based on its anti-apoptotic and anti-inflammatory effects. Current research indicates that FMN can suppress the expression of activator protein-1 (AP-1) [19]. AP-1 is a transcription factor composed of protein dimers, primarily from the Jun and Fos protein families. It plays a crucial role in various biological processes (BPs), including apoptosis and inflammation [20]. We investigated the protective effects of FMN in a GES-1 cell model treated with 40 μM N-methyl-N^’^-nitro-N-nitrosoguanidine (MNNG). Furthermore, we explored the potential mechanisms of FMN treatment in this MNNG-triggered CAG model.

CAG presents a complex pathogenesis involving multiple interconnected pathways, including inflammatory responses, apoptotic regulation, and epithelial remodeling. Simultaneously, FMN, as a natural isoflavone, exhibits polypharmacological characteristics with the potential to modulate diverse molecular targets. Given the multi-pathway nature of CAG pathogenesis and the polypharmacological properties of FMN, network pharmacology offers a valuable methodological approach for characterizing their potential interactions. Network pharmacology is utilized to elucidate the complex interactions among drugs, diseases, and therapeutic targets. This approach enables researchers to identify and predict the effective components and therapeutic targets of drugs. Network pharmacology provides valuable insights for drug repurposing and the discovery of new indications [21,22]. Ye *et al*. applied network pharmacology to predict the biological targets underlying quercetin's therapeutic efficacy in treating non-small cell lung cancer [23]. Qian *et al*. utilized network pharmacology to demonstrate that FMN mitigates β-adrenergic receptor-mediated cardiac fibrosis by enhancing mitochondrial function [24]. Consequently, the present study employed network pharmacology analysis to identify the relevant BPs and potential targets of FMN in the treatment of CAG. Preliminary validation was subsequently conducted through *in vitro* experiments, thereby establishing a foundation for further research. The overall study design is presented in Fig. (**1**).

## 2 MATERIALS AND METHODS

### 2.1 Network Pharmacology Studies

Potential therapeutic targets of FMN for CAG and their underlying mechanisms were systematically predicted through integrated network pharmacology approaches combining target prediction, protein-protein interaction (PPI) network analysis, and functional enrichment. Table **1** summarizes all data sources and computational tools employed in this study.

#### 2.1.1 Acquisition of Overlapping Targets between Gene Targets of FMN and CAG

The associated genes of FMN were obtained from the STP database and the HERB database. The term “chronic atrophic gastritis” was utilized as a search query to extract human genes from the GeneCards, OMIM, and PharmGkb databases for the prediction of CAG-related genes. The overlapping targets between FMN-associated genes and CAG-related genes were identified and visualized through Venn diagram analysis.

#### 2.1.2 Creation of the PPI Network

Utilizing STRING database [25], this investigation integrated the overlapping targets of FMN and CAG to obtain PPI data. A medium-confidence threshold (interaction score ≥0.4) was applied, with *Homo sapiens* as the specified organism, and disconnected nodes were hidden in the network. The interaction network was constructed using Cytoscape v3.9.1 software with imported STRING-derived PPI data. The CytoNCA [26] plugin was used to calculate degree centrality, and hub proteins (high-degree nodes) were identified, potentially serving as critical pharmacological targets or regulatory nodes.

#### 2.1.3 Bioinformatic Enrichment Analyses of Gene Ontology (GO) Terms and Reactome Pathways

To elucidate the functions of overlapping genes between FMN and CAG, the evaluation of biological process (BP), molecular function (MF), and cellular component (CC) enrichment was performed using GO enrichment analysis. Reactome pathway analysis further elucidated potential mechanisms of FMN treatment in CAG. All analyses were conducted using the DAVID database, with corrected *P* < 0.05 considered statistically significant. The significantly enriched results were visualized using the SRplot online platform.

### 2.2 Cell Line and Cell Culture

Human gastric epithelial cells (GES-1; BNCC337969; BeNa Culture Collection, China) were cultured in RPMI-1640 medium (C11875500BT; Gibco, USA) supplemented with 10% fetal bovine serum (10099141; Gibco, USA), 1% penicillin (MK008A; BIOMIKY, China), and streptomycin (MK008A; BIOMIKY, China) at 37°C in a humidified 5% CO_2_ atmosphere.

### 2.3 Cell Viability Assay

Cell viability was detected using the CCK-8 kit (CK04; Dojindo Laboratories, Japan). In a 96-well plate, GES-1 cells were cultured at a density of 5 × 10^3^ cells per well. Various experimental groups and dosages were established in accordance with the experimental objectives. To establish the optimal MNNG (M105583; Aladdin, China) concentration for CAG model induction, a range of MNNG concentrations (5, 10, 20, 40, 80, and 160 μM) was applied to cells in the logarithmic growth phase for 24 hours. Based on previously established criteria [27], we defined 60% cell survival as the optimal threshold for model establishment. Our experimental data demonstrated that 40 μM MNNG treatment for 24 hours achieved this target survival rate while producing the most favorable pathophysiological outcomes. To identify safe concentrations of FMN for subsequent experiments, GES-1 cells were treated with varying concentrations of FMN (1.25, 2.5, 5, 10, 20, 40, 60, 80, and 100 μM) for 24 hours, and cell viability was measured. Cells were pretreated with different concentrations of FMN (1.25, 2.5, 5, 10, 20, 40, and 60 μM) for 24 hours, followed by co-incubation with 40 μM MNNG for an additional 24 hours. Based on these preliminary results, FMN concentrations of 5, 10, and 20 μM were selected for further experiments. In subsequent assays, cells were pretreated with FMN (5, 10, or 20 μM) for 24 hours before exposure to 40 μM MNNG for another 24 hours. In a separate experiment, cells were treated with 10 μM T-5224 and 20 μM FMN for 24 hours prior to 40 μM MNNG exposure. All procedures were conducted under light-protected conditions. After treatments, 10 μL of CCK-8 reagent was added to each well, and the absorbance at 450 nm was measured following a 2-hour incubation. Each condition included 6 replicate wells.

### 2.4 ELISA

The concentrations of IL-6 (GEH0001-96T), IL-1β (GE H0002-96T), IL-10 (GEH0003-96T), and TNF-α (GEH0 004-96T) in the supernatants from GES-1 cells were quantified using ELISA kits (Servicebio, Wuhan, China). All procedures were carried out as instructed by the manufacturer.

### 2.5 Western Blotting Analysis

The Column Tissue and Cell Protein Extraction Kit (PC201; EpiZyme, China) was employed to extract total proteins. The protein samples were separated by 12% SDS-PAGE electrophoresis and subsequently transferred onto polyvinylidene difluoride membranes. After blocking with the Western Blocking buffer (BL535A; Biosharp, China) for 2 hours at room temperature (RT), the membranes were incubated for 12 hours at 4°C with primary antibodies for β-actin (GB15003-100; Servicebio, China; 1:2000), c-Jun (A0246; ABclonal, China;1:250), nuclear receptor coactivator 1 (NCOA1,2859; Abcam, UK; 1:200), and c-Fos (ab222699; Abcam, UK; 1:200), which was followed by a 2-hour incubation at RT with horseradish peroxidase-conjugated secondary antibodies (GB23303; Servicebio, Wuhan, China; 1:10000) with gentle agitation. Protein bands were visualized using enhanced chemiluminescence (ECL) substrate (BL520B; Biosharp, China) and quantified using ImageLab software (Bio-Rad, USA).

### 2.6 Cell Apoptosis Assay

To quantify cell apoptosis, the Annexin V-APC/PI Apoptosis kit (E-CK-A217; Elabscience, Wuhan, China) was utilized. Following two washes with ice-cold PBS, the cells were subsequently transferred to 500 µL of binding buffer. Then, the cells were exposed to 5 µL Annexin V-APC and 5 µL of propidium iodide in the dark at RT for 5 minutes. The cell apoptosis was measured using a flow cytometer (Beckman Coulter, USA) within 1 hour, and the data were analyzed using FlowJo software (FlowJo LLC, USA).

### 2.7 Immunofluorescence Staining

GES-1 cells were fixed with 1% paraformaldehyde (BL9 08A; Biosharp, China) for 30 minutes and permeabilized with 0.5% Triton X-100 (G1204-100ML; Servicebio, Wuhan, China) for 10 minutes. Then, the GES-1 cells were incubated with PBS containing 1% BSA for 1 hour. The immunofluorescence staining was conducted using c-Jun (1:100), c-Fos (1:100), and NCOA1 (1:100) antibodies overnight at 4°C. A secondary antibody coupled with Alexa Fluor 555 (1:2000) was applied for 2 hours at RT. 4′,6-diamidino-2-phenylindole (1 mg/mL) was utilized to stain the nuclei. The fluorescence intensity was measured using a fluorescence microscope (DMi8; Leica Microsystems, Germany).

### 2.8 Statistical Analysis

The experimental values have been presented as mean ± SD, which were analyzed using GraphPad Prism 10.0 software (San Diego, CA, USA). To compare multiple datasets, one-way ANOVA with Tukey post-hoc test was employed. The significance was determined at *P* < 0.05.

## 3 RESULTS

### 3.1 Network Pharmacology Analyses

#### 3.1.1 Targets of FMN Intersecting with CAG

As depicted in Fig. (**2A**), 1094 CAG-related targets and 84 potential FMN-related targets were identified from internet databases. Additionally, 21 overlapping targets of FMN in CAG treatment were identified by Venn diagram analysis. The overlapping targets might act as potential therapeutic targets for FMN in CAG therapy.

#### 3.1.2 Analysis of the PPI Network and Identification of Core Targets

**Fig. (2):**
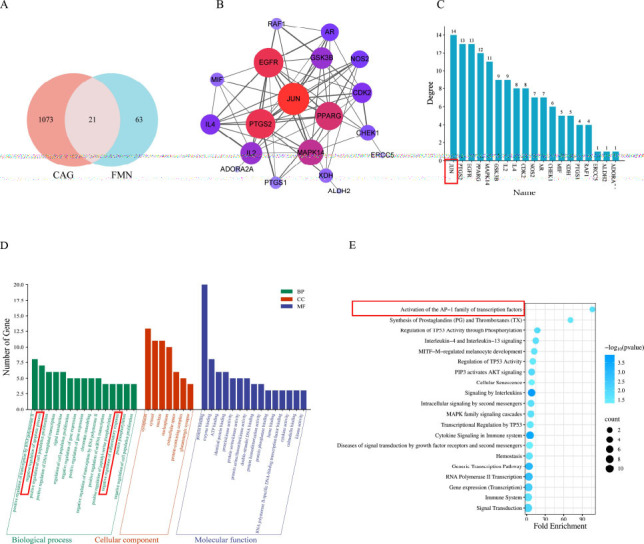
Network pharmacology analyses. (**A**) Venn diagram of overlapping targets between FMN and CAG. (**B**) PPI network of FMN against CAG. (**C**) The top 19 core targets in the PPI network. (**D**) The top 15 terms of biological function, including BP, CC, and MF, based on GO enrichment analysis. (**E**) The bubble chart of the 20 pathways based on Reactome enrichment analysis.

Core genes and critical functional modules were identified using the STRING database through PPI network analysis, as previously described [28]. Filtration of outcomes was conducted with a minimum interaction score of ≥ 0.4 The overlapping targets were further analyzed for PPI network architecture in the STRING database to detect core targets. The PPI network study resulted in 19 nodes and 69 edges, as observed using Cytoscape 3.9.1 software (Fig. **2B**). Based on degree value, the top 5 pertinent targets of FMN against CAG were JUN, PTGS2, EGFR, PPARγ, and MAPK14, respectively. JUN exhibited the highest degree of centrality among all identified targets (Fig. **2C**; Supplementary Table **S1**).

#### 3.1.3 GO and Reactome Pathway Analyses

We used the enrichment analysis of GO and Reactome pathway to examine the 21 overlapping targets in order to elucidate the functional annotations and principal signaling pathways associated with the target proteins [29]. The extent and statistical significance of gene enrichment have been indicated by gene count, fold enrichment, and *p-*value, respectively. 119 GO items were collected in line with the screening criteria (*P* < 0.05). A total of 68 terms were related to BP, with 7 terms pertaining to CC and 44 terms associated with MF. The 15 most representative GO keywords were visualized (Fig. **2D**). BP encompassed the negative regulation of the inflammatory response, negative regulation of the apoptotic process, and transcription by RNA polymerase II, among others. MF comprised protein binding, enzyme binding, protein homodimerization activity, and cytokine activity, among others. In terms of CC, the cytoplasm, cytosol, and nucleus were notably enriched. The screening criteria revealed 20 pathways to be significantly enhanced (*P* < 0.05). Fig. (**2E**) displays the 20 pathways selected from the Reactome database. The findings revealed the pathways to be enriched in the activation of the AP-1 family transcription factors, regulation of TP53 activity, PIP3-activated AKT signaling, and interleukin signaling. Based on its highest fold enrichment among all identified pathways, the AP-1 signaling was prioritized for experimental validation.

### 3.2 FMN Mitigated MNNG-triggered Decrease in GES-1 Cell Viability

**Fig. (3):**
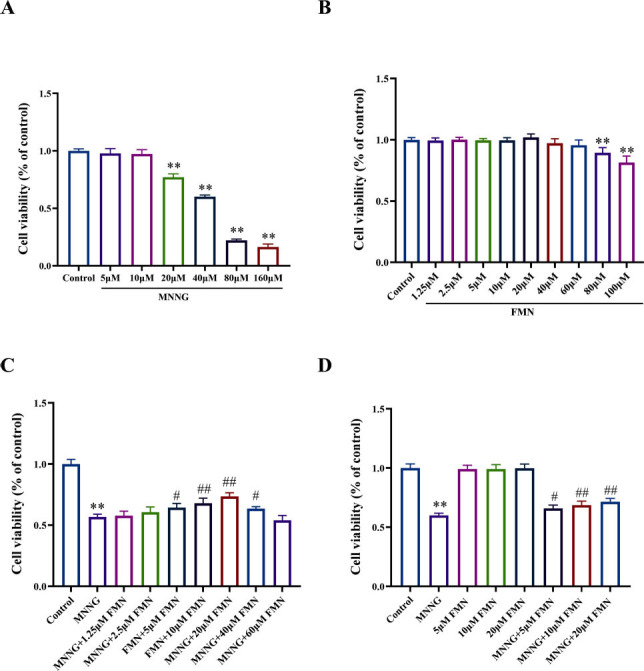
FMN mitigated the MNNG-triggered decrease in GES-1 cell viability. (**A**) Effects of different doses (0, 5, 10, 20, 40, 80, and 160 μM) of MNNG on the viability of GES-1 cells. (**B**) Effects of different concentrations (1.25, 2.5, 5, 10, 20, 40, 60, 80, and 100 μM) of FMN on the viability of GES-1 cells for 24 hours. (**C**) Protective effect of FMN (1.25, 2.5, 5, 10, 20, 40, and 60 μM) pretreatment on the viability of MNNG-triggered GES-1 cells. (**D**) Cell viability with escalating FMN doses (5, 10, and 20 μM) following 40 μM MNNG exposure. Values are expressed as mean ± SD (*n* = 6). *P < 0.05, **P < 0.01 compared to the control group; ^#^*P* < 0.05, ^##^*P* < 0.01 compared to the MNNG group.

Based on the network pharmacology predictions, we first evaluated the cytotoxic effects of MNNG and the therapeutic potential of FMN. After being exposed to MNNG at 0, 5, 10, 20, 40, 80, and 160 μM for 24 hours, the viability of GES-1 cells was assessed. When different doses of MNNG were subjected to GES-1 cells, the cell viability significantly reduced in a concentration-dependent way (Fig. **3A**). The cellular model of CAG was produced by treating GES-1 cells with 40 μM MNNG for 24 hours, which led to a 60% survival rate. We next examined how different FMN concentrations (1.25, 2.5, 5, 10, 20, 40, 60, 80, and 100 μM) affected GES-1 cell viability. Results showed no significant impact on viability at concentrations ≤ 60 μM (Fig. **3B**). To determine the optimal protective concentration of FMN, GES-1 cells were pretreated with different FMN concentrations (1.25, 2.5, 5, 10, 20, 40, and 60 μM) for 24 hours before MNNG exposure. The CCK-8 assay revealed that 5, 10, 20, and 40 μM FMN exerted protective effects on MNNG-triggered GES-1 cells, with 20 μM FMN providing the strongest protection (Fig. **3C**). The viability of GES-1 cells was not significantly affected by FMN at 5, 10, and 20 μM concentrations. Additionally, the experimental results from the CCK-8 assay showed that FMN (5, 10, and 20 μM) restored the reduced viability of GES-1 cells caused by MNNG (Fig. **3D**).

### 3.3 FMN Relieved MNNG-triggered Inflammatory Response and Apoptosis in GES-1 Cells

**Fig. (4):**
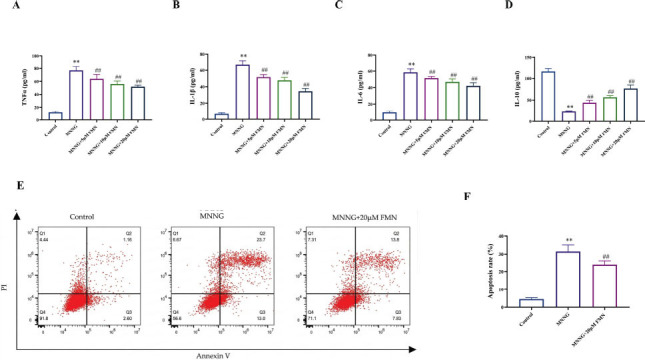
FMN mitigated the inflammatory response and apoptosis induced by MNNG in GES-1 cells. Prior to exposure to 40 μM MNNG, GES-1 cells were pretreated with a range of FMN concentrations. (**A-D**) Detection of TNF-α, IL-10, IL-1β, and IL-6 expression by ELISA kit. (E-F) Apoptosis of GES-1 cells by flow cytometry analysis. Values are expressed as mean ± SD (*n*=6 for A-D; *n*=4 for **E** and **F**). *P < 0.05, **P < 0.01 compared to the control group; ^#^*P* < 0.05, ^##^*P* < 0.01 compared to the MNNG group.

The levels of inflammatory cytokines were detected to estimate the inflammatory reaction of GES-1 cells. ELISA showed that exposure to MNNG led to an elevation in proinflammatory cytokines, including TNF-α, IL-6, and IL-1β levels, while decreasing the expression of IL-10. In GES-1 cells subjected to MNNG, FMN diminished the expression of the proinflammatory cytokines and enhanced IL-10 expression concentration-dependently (Fig. **4A-D**). MNNG treatment significantly induced an apoptosis rate of 31.48% ± 3.68% in GES-1 cells. FMN (20 µM) was found to dramatically alleviate MNNG-triggered apoptosis, and the apoptosis rate reduced to 24.22% ± 2.09% in GES-1 cells (Fig. **4E-F**).

### 3.4 FMN Inhibited NCOA1/AP-1 Signaling Axis in MNNG-triggered GES-1 Cells

**Fig. (5):**
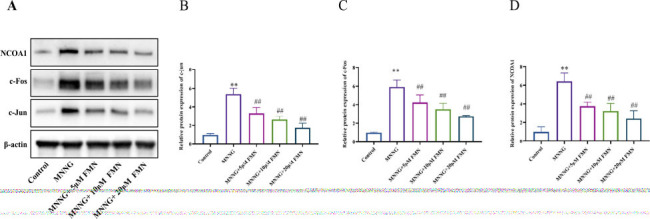
(**A**) Western blot images of NCOA1, c-Fos, and c-Jun protein level. (**B-D**) Relative NCOA1, c-Fos, and c-Jun expression levels in GES-1 cells. Values are expressed as mean ± SD (*n* = 4). *P < 0.05, **P < 0.01 compared to the control group; ^#^*P* < 0.05, ^##^*P* < 0.01 compared to the MNNG group.

Based on earlier network pharmacology analysis outcomes, the AP-1 signaling was selected for experimental validation. NCOA1 has been reported to be overexpressed in gastric cancer tissues [30]. NCOA1 was implicated in the transcriptional regulation of genes through its interactions with other transcription factors. It has been reported that NCOA1 functions as a transcriptional coactivator for the AP-1 transcription factor, and their collaborative action promotes tumorigenesis [31]. As CAG is the initial stage of precancerous lesions in GC, and AP-1 is strongly influenced in tumorigenesis [32, 33], we hypothesized that FMN may provide a therapeutic effect in CAG by reducing inflammation and cell death *via* regulating the NCOA1/AP-1 signaling axis. GES-1 cells were pretreated with FMN (5, 10, and 20 µM), and subsequently stimulated with 40 µM of MNNG for 24 hours. Western blot analysis revealed that MNNG exposure increased the protein expression of NCOA1, c-Jun, and c-Fos in GES-1 cells. FMN resulted in a concentration-dependent downregulation of NCOA1, c-Jun, and c-Fos expression in MNNG-triggered GES-1 cells (Fig. **5**). As illustrated in Fig. (**6**), to detect NCOA1, c-Jun, and c-Fos expressions and locations, immunofluorescence staining of GES-1 cells in all groups was applied. NCOA1, c-Jun, and c-Fos expression was primarily expressed in the nuclei, and FMN mitigated the elevation of NCOA1, c-Jun, and c-Fos expression after MNNG treatment.

### 3.5 Blocking AP-1 Activation Enhanced the Protective Effect of FMN in MNNG-triggered GES-1 Cells

**Fig. (7):**
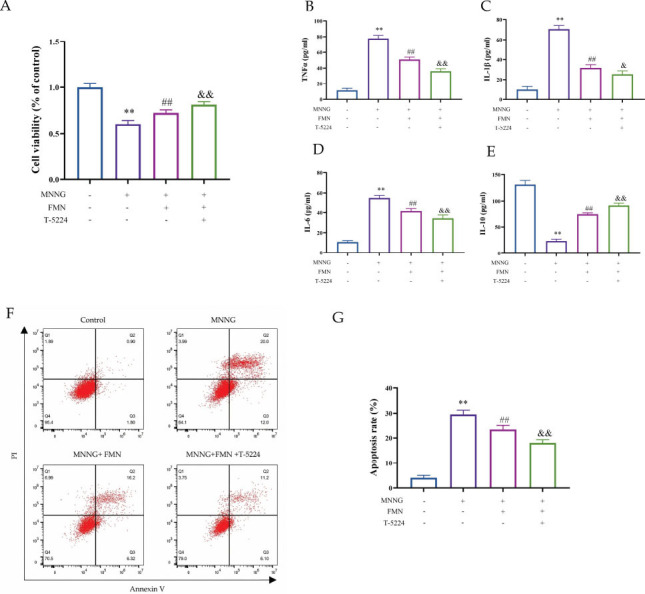
Blocking AP-1 activation enhanced the protective impact of FMN on MNNG-treated GES-1 cells. GES-1 cells in different groups were pretreated with 20 μM FMN or with a combination of 20 μM FMN and 10 μM T-5224 for 24 hours before exposure to 40 μM MNNG. (**A**) The viability of GES-1 cells in different groups. (**B-E**) TNF-α, IL-10, IL-1β, and IL-6 levels in culture medium. (**F, G**) Cell apoptosis in different groups. Values are expressed as mean ± SD (*n*=6 for A-E; *n*=4 for F and G). *P < 0.05, **P < 0.01 compared to the control group; ^#^*P* < 0.05, ^##^*P* < 0.01 compared to the MNNG group; ^&^*P* < 0.05, ^&&^*P* < 0.01 compared to the MNNG + FMN group.

FMN at a concentration of 20 µM exerted the most significant effects. Hence, it was selected for the subsequent tests. To elucidate the putative downstream pathway of FMN in the MNNG-stimulated cellular model of CAG linked with inflammation and apoptosis, the AP-1 inhibitor T-5224 was used. The CCK-8 assay indicated that, in comparison to FMN treatment alone, T-5224 combined with FMN enhanced the viability of GES-1 cells (Fig. **7A**). T-5224 treatment reduced TNF-α, IL-6, and IL-1β levels, and elevated IL-10 level in the cell supernatant (Fig. **7B-E**). The apoptosis rate of FMN-treated GES-1 cells was 23.34% ± 1.62%, while the combination of FMN and T-5224 reduced the apoptosis rate to 18.07% ± 1.23%. As depicted, T-5224 enhanced the inhibitory effect of FMN against MNNG-triggered cell apoptosis in GES-1 cells (Fig. **7F, G**).

## 4 DISCUSSION

CAG is a digestive disorder distinguished by complex, refractory, and varied etiologies [34]. One of the primary causes of CAG is exposure to N-nitroso compounds [2]. MNNG, an alkylating agent, is frequently employed to simulate the mutagenic and carcinogenic effects of N-nitroso compounds [35]. The GES-1 cell line, derived from human gastric mucosal cells, has been widely used in studies on carcinogenesis due to its non-tumorigenic characteristics and immortal phenotype [36]. The GES-1 cells triggered by MNNG mimic the progression from normal gastric mucosa to CAG and, ultimately, GC [37]. The pathogenic mechanism of MNNG-triggered CAG encompasses inflammation-related and apoptosis-related signaling pathways, including NF-κB [38], MAPK [39], and HIF-1α [40]. Consequently, our research work adopted MNNG to trigger damage in GES-1 cells, simulating the pathological process of alkylating agent injury to the gastric mucosa, thus resulting in CAG.

Inflammation elicits an immune response that aids in pathogen clearance and tissue healing. However, cytokines produced from chronic inflammation can potentially facilitate cancer formation through various routes [41]. CAG is primarily defined by inflammatory infiltration and cellular destruction [42]. The change from gastric mucosal damage to metaplasia is initiated by inflammatory cell infiltration and cytokine production [43]. The pro-inflammatory cytokines we detected in the present study, including IL-1β, IL-6, and TNF-α, have been found to be essential contributors to the progression of CAG [38, 44-46]. Inhibiting inflammatory processes to interrupt the aforementioned progression could prevent the advancement of CAG to intestinal metaplasia and, ultimately, to GC [47]. Another pathophysiological feature of CAG is cellular destruction, which is directly associated with apoptosis. Recent research has shown strong evidence linking CAG progression to apoptosis. Targa *et al*. reported that the apoptotic index of gastric mucosa in CAG tissues was markedly elevated compared to that in normal tissues [48]. Immunohistochemical studies of clinical gastrectomy tissues have shown an increase in apoptotic gastric epithelium to be associated with CAG and intestinal metaplasia [49]. Our results indicated that MNNG induced apoptosis in GES-1 cells, being consistent with the literature that CAG leads to apoptosis in gastric mucosal cells. This suggests that apoptosis is an important intervention target for CAG. Therefore, drugs with anti-inflammatory and anti-apoptotic features may have potential effects in the treatment of diseases, like CAG [2].

Recent studies have shown that FMN exerts potential therapeutic effects by modulating inflammatory responses and apoptosis. For instance, the expression of proinflammatory cytokines, including IL-1β, IL-6, and TNF-α, was downregulated by FMN in primary cultured chondrocytes after IL-1β treatment [50]. FMN mitigated the neurological impairment and pathological injury in the brain tissues of an ischemia-reperfusion rat model. It was found that FMN reduced the area of cerebral infarction and inhibited neural cell apoptosis [51]. Additionally, FMN was non-genotoxic and exhibited marked antimutagenic activity *in vitro*, indicating its potential to be employed as a therapeutic medication for disorders related to genomic instability [52]. Consequently, FMN was utilized in this investigation for its anti-inflammatory and anti-apoptotic properties. The effectiveness of FMN was initially confirmed in our study on GES-1 cells. FMN markedly enhanced the vitality of GES-1 cells cultured with MNNG, while diminishing their inflammation and apoptosis levels. These findings demonstrated that FMN possesses a therapeutic benefit for CAG caused by MNNG. Currently, the main treatment for CAG includes acid suppression, gastric mucosal protection, and *Helicobacter pylori* eradication. The anti-inflammatory and anti-apoptotic effects of FMN may serve as a complementary therapeutic mechanism for the treatment of CAG.

Network pharmacology analysis revealed FMN to modulate 21 targets implicated in CAG. Further analysis of PPI networks identified 19 core targets, with c-Jun emerging as the most central target in FMN-mediated CAG treatment, characterized by the highest degree. The BP modulated by FMN in the context of CAG encompassed the negative regulation of apoptosis and inflammatory responses, both of which are intricately linked to the pathogenesis of CAG. Thereafter, pathway enrichment analysis highlighted AP-1 signaling as the predominant pathway. FMN exerted protective effects *via* the regulation of AP-1 signals in CAG. C-Jun is a basic leucine zipper transcription factor that functions as either a homodimer or heterodimer, regulating gene transcription *via* binding to DNA [53-57]. AP-1 is a dimeric transcription factor consisting of subunits, including c‐Jun and c‐Fos [58]. Growth factors, cytokines, or infections stimulate AP-1 activation, which subsequently modulates gene transcription, thereby influencing physiological processes, such as cell proliferation, transformation, inflammation, and apoptosis [59, 60]. Research indicates that the activation of AP-1 raises the level of inflammation, and inhibition of AP-1 has therapeutic effects in controlling inflammation [61]. Suppression of c-Jun and c-Fos could mitigate gastric mucosal damage caused by *Helicobacter pylori* infection [62], and upregulation of AP-1 is associated with the origin and progression of GC cells [63]. Concurrently, studies have demonstrated transcriptional activation of AP-1 to exert a regulatory effect on cell apoptosis. Monotropein has been shown to mitigate cell apoptosis through the downregulation of AP-1 signaling [64]. AP-1 inhibition has been identified as a protective measure for ventricular cardiomyocytes against hypertrophy and apoptosis [65].

NCOA1 is a member of the p160 family. It is a transcriptional coactivator for other transcription factors, thereby regulating the transcriptional activity of target genes. It has also been implicated in carcinogenic processes across various cancers [66-68]. Previous research has demonstrated that the upregulation of c-Jun, c-Fos, and NCOA1 contributes to the pathogenesis of CAG and GC. As a carcinogen, MNNG efficiently promoted the expression of c-Fos and c-Jun, correlating with a dose-dependent rise in the AP-1 activity [69]. It was reported that AP-1, CREB, and NF-κB-driven reporter genes were significantly increased in MNNG-treated cells [70]. *Helicobacter pylori* infection increased the expressions of AP-1, c-Jun, and c-Fos in a mouse cancer model [71]. Meng *et al*. found GC tissues to have elevated NCOA1 expression, and higher overall survival rates of patients with GC were associated with a lower NCOA1 expression. Data from the GEO and Oncomine datasets showed NCOA1 to be a reliable predictor for GC outcomes [72].

Studies have shown FMN to exert biological protection by downregulating AP-1. Through modulating the AP-1 signal, FMN reduced osteoclast differentiation and calcium loss in mice with type 1 diabetes [19]. FMN demonstrated protective activity in knee joints by simultaneously targeting both NF-κB and MAPK signaling cascades [73]. Our study indicated c-Jun to be a significant target of FMN, implying AP-1 to be a potential therapeutic target for mitigating MNNG-related CAG. However, how FMN affects CAG progression through AP-1 is unclear. The present study, for the first time, identified that FMN inhibited NCOA1 in the CAG cell model, indicating that NCOA1 may be a novel regulator in the process. NCOA1 has a vital function in enhancing the proliferation of different malignancies by interacting with nuclear receptors and other transcription factors (including AP-1, Est-2, PEA3, β-catenin, and SMAD) to activate gene expression [30]. It was reported that NCOA1 coactivated AP-1 and mediated its transcription by interacting with the subunits of c-Jun and c-Fos [74]. Together with AP-1, NCOA1 stimulated the expression of ITGA5 and accelerated the capability of breast cancer cells to adhere to fibronectin [75]. Additionally, NCOA1-knockdown suppressed TPA-induced AP-1 transcriptional activity [76]. According to our data, NCOA1 affected inflammation and apoptosis in the CAG cell model *via* AP-1 signal, indicating the transcriptional activity of AP-1 to be a regulating target of NCOA1. The evidence may reveal the underlying mechanism of FMN for AP-1 modulation in the CAG cell model.

Our data revealed c-Jun, c-Fos, and NCOA1 protein levels to be increased in MNNG-triggered GES-1 cells. FMN downregulated the protein levels of c-Jun, c-Fos, and NCOA1. A selective AP-1 inhibitor, T-5224, enhanced the effects of FMN on cell viability, inflammatory response, and apoptosis in MNNG-triggered CAG cells. These findings revealed that FMN exerted a protective effect against the major pathological processes of CAG *via* the regulation of the NCOA1/AP-1 signaling axis. Recent studies have demonstrated multiple natural compounds to exhibit significant preventive and therapeutic effects against CAG [77]. Among them, berberine has shown promising therapeutic effects in various CAG models [27, 78, 79]. Both FMN and berberine have been reported to significantly reduce the levels of pro-inflammatory cytokines, including TNF-α, IL-6, and IL-1β, and effectively alleviate MNNG-induced inflammatory damage as well as improve the survival rate of GES-1 cells. However, their mechanisms of action are distinct. Berberine primarily ameliorates inflammation by activating the TGF-β1/PI3K signaling pathway and exerts therapeutic effects in CAG by promoting cellular autophagy [27]. FMN modulates the NCOA1/AP-1 signaling axis, simultaneously targeting both inflammatory responses and apoptotic processes. On the other hand, mechanistic connections exist between these two compounds. For instance, autophagy efficiently removes dysfunctional or damaged organelles, thereby reducing cellular degeneration, suppressing inflammatory responses, and preventing aberrant apoptosis [80]. Although their mechanisms differ, both compounds ultimately attenuate inflammation, enhance cell survival, and maintain cellular homeostasis, which contributes to their therapeutic effects against CAG.

## CONCLUSION

In conclusion, FMN mitigated the pathological processes of inflammation and apoptosis triggered by MNNG in the progression of CAG. The primary mechanism involved the suppression of inflammation and apoptosis by the NCOA1/AP-1 signaling axis. The present study may offer pharmacological evidence for FMN, paving the way for research on novel treatment against CAG.

## STUDY LIMITATIONS

Nonetheless, this study has involved some limitations. The mechanism by which the coactivation of NCOA1 and AP-1 contributes to the pathophysiology of CAG needs to be examined. The therapeutic effects of FMN in CAG require validation through animal studies. Future research will explore additional signaling pathways potentially mediated by FMN in CAG and will include comparative studies with existing drugs or natural compounds currently used for CAG treatment.

## Figures and Tables

**Fig. (1):**
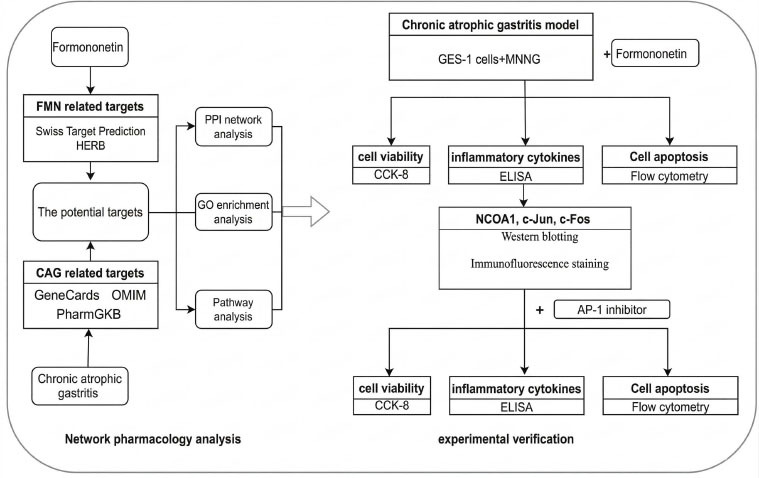
Study workflow combining network pharmacology prediction analysis and *in vitro* experimental validation.

**Fig. (6):**
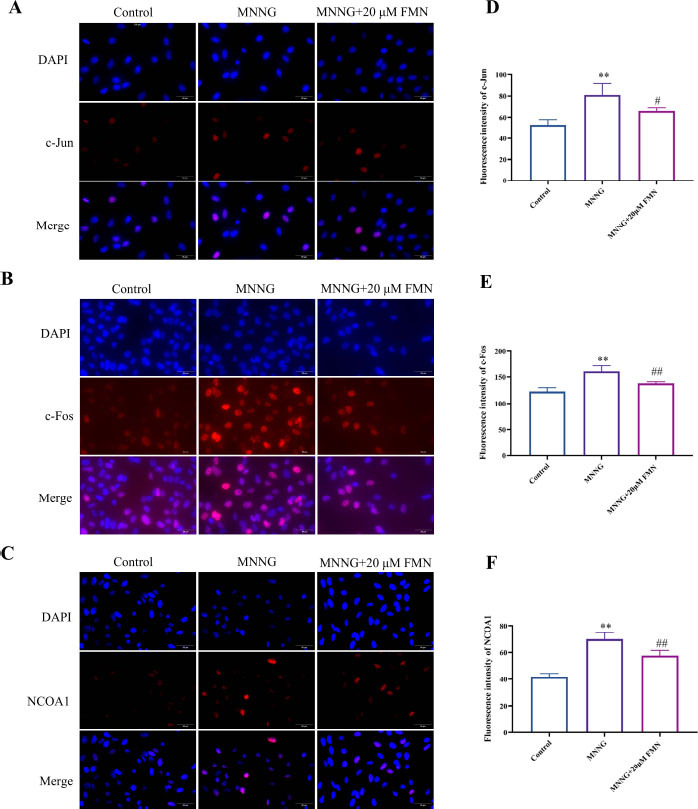
(**A-C**) Immunofluorescence staining of c-Jun, c-Fos, and NCOA1 in GES-1 cells (Scale bar: 50μm). (**D-F**) Mean fluorescence intensity of c-Jun, c-Fos, and NCOA1. Values are expressed as mean ± SD (*n* = 5). *P < 0.05, **P < 0.01 compared to the control group; ^#^*P* < 0.05, ^##^*P* < 0.01 compared to the MNNG group.

**Table 1:** All data sources and computational tools employed in this study.

**Name**	**Full Name**	**The url**
STP	Swiss Target Prediction	http://www.swisstargetprediction.ch/
HERB	High-throughput Experiment-and Reference-guided Batabase of Traditional Chinese Medicine	http://herb.ac.cn/
OMIM	Online Mendelian Inheritance in Man	https://www.omim.org/
GeneCards	GeneCards: The Human Gene Database	https://www.genecards.org/
PharmGKB	Pharmacogenomics Knowledge Base	https://www.pharmgkb.org/
STRING	Search Tool for the Retrieval of Interacting Genes/Proteins	https://string-db.org/
DAVID	Database for Annotation, Visualization, and Integrated Discovery	https://david.ncifcrf.gov/
SRplot	-	https://www.bioinformatics.com.cn/srplot
Cytoscape3.9.1	Cytoscape 3.9.1 - Network Visualization and Analysis Software	-
CytoNCA	Cytoscape Network Centrality Analysis	-

## Data Availability

Not applicable.

## LIST OF ABBREVIATIONS

AP-1: Activator Protein-1
BP: Biological Process
CAG: Chronic Atrophic Gastritis
CC: Cellular Component
ECL: Enhanced Chemiluminescence
FMN: Formononetin
GC: Gastric Cancer
GES-1: Human Gastric Epithelial Cells
GO: Gene Ontology
MF: Molecular Function
MNNG: N-methyl-N'-nitro-N-nitrosoguanidine
NCOA1: Nuclear Receptor Coactivator 1
PPI: Protein-protein Interaction
RT: Room Temperature
