# Supplementary material for: Formononetin Alleviates MNNG-Triggered Chronic Atrophic Gastritis: Its Potential Mechanisms
Source: Comb Chem High Throughput Screen. 2025 Jul 3;29(4):717–30. doi: 10.2174/0113862073409559250618212035 (PMC13312389; doi:10.2174/0113862073409559250618212035)
Supplement: Supplementary file 1 [file CCHTS-29-4-717_SD1.pdf]

Supplementary Material

Formononetin Alleviates MNNG-Triggered Chronic Atrophic Gastritis: Its Potential Mechanisms

Yuling Wei[1], Wenhui Wu[1], Min Duan[2], Ting Li[1], Mei Liu[1] and Jinyan Li[3,*]

[1]*Department of Pharmacy, Chongqing Hospital of Traditional Chinese Medicine, Chongqing 400000, China;* [2]*Department of Gastroenterology, Chongqing Hospital of Traditional Chinese Medicine, Chongqing 400000, China.* [3]*Department of Obstetrics and Gynecology, Chongqing Hospital of Traditional Chinese Medicine, Chongqing 400000, China*

Supplementary table S1. Core targets and topological parameters.

Name	Degree Centrality (DC)	Betweenness Centrality (BC)	Closeness Centrality (CC)	LAC	Eigenvector Centrality (EC)
JUN	14	52.96666666666667	0.8181818181818182	6.857142857142857	0.35505908727645874
PTGS2	13	28.199999999999996	0.75	6.769230769230769	0.34087127447128296
EGFR	13	31.800000000000004	0.75	6.615384615384615	0.33538225293159485
PPARG	12	21.9	0.72	6.666666666666667	0.325035959482193
MAPK14	11	17	0.6923076923076923	6.545454545454546	0.30725958943367004
IL2	9	35.63333333333333	0.6206896551724138	5.333333333333333	0.2475471943616867
GSK3B	9	6.433333333333334	0.6206896551724138	6	0.2582314610481262
IL4	8	1.6333333333333333	0.6	6	0.24496738612651825
CDK2	8	2.9333333333333336	0.6	6	0.24620269238948822
NOS2	7	4	0.6	5.142857142857143	0.20961222052574158
AR	7	1.5	0.5625	5.142857142857143	0.21273480355739594
CHEK1	6	34	0.5625	3.3333333333333335	0.16281460225582123
XDH	5	34	0.5454545454545454	2.4	0.1335231512784958
MIF	5	0	0.5454545454545454	4	0.16341839730739594
RAF1	4	0	0.5142857142857142	3	0.12442541867494583
PTGS1	4	0	0.5142857142857142	3	0.14029090106487274
ERCC5	1	0	0.3673469387755102	0	0.01742502488195896
ALDH2	1	0	0.36	0	0.014335857704281807
ADO-RA2A	1	0	0.391304347826087	0	0.026570498943328857
